# Clinical Significance of Rare Non-*Candida* Yeasts in Pediatric Fungemia: A Retrospective Analysis

**DOI:** 10.3390/jof12040235

**Published:** 2026-03-25

**Authors:** Gül Arga, Halil Özdemir, Duygu Öcal, Elif Somuncu, Hülya Akat, Döndü Nilay Penezoğlu, Hatice Belkıs İnceli, Yasemin Ezgi Köstekçi, Hasan Fatih Çakmaklı, Merve Havan, Sonay İncesoy Özdemir, Tanıl Kendirli, Mehmet Ertem, Nurdan Taçyıldız, Ergin Çiftçi

**Affiliations:** 1 Department of Pediatric Infectious Diseases, Ankara University Faculty of Medicine, Ankara 06590, Turkey; doktorhalil@gmail.com (H.Ö.); eliffcobann@gmail.com (E.S.); hulya.akatt@gmail.com (H.A.); nilay.217217@gmail.com (D.N.P.); belkisinceli@gmail.com (H.B.İ.);; 2 Department of Medical Microbiology, Ankara University Faculty of Medicine, Ankara 06590, Turkey; drduygunil@gmail.com; 3 Department of Neonatology, Ankara University Faculty of Medicine, Ankara 06590, Turkey; ezgikostekci@gmail.com; 4 Department of Pediatric Hematology, Ankara University Faculty of Medicine, Ankara 06590, Turkey; hasanfath@yahoo.com (H.F.Ç.); mertem2004@yahoo.com (M.E.); 5 Department of Pediatric Intensive Care, Ankara University Faculty of Medicine, Ankara 06590, Turkey; merve.havan@gmail.com (M.H.); tanilkendirli@hotmail.com (T.K.); 6 Department of Pediatric Oncology, Ankara University Faculty of Medicine, Ankara 06590, Turkey; sincesoy@yahoo.co.uk (S.İ.Ö.); nurdantacyildiz@yahoo.com (N.T.)

**Keywords:** fungemia, non-*Candida* yeasts, antifungal treatments, *Trichosporon asahii*, *Magnusiomyces clavatus*, *Rhodotorula mucilaginosa*

## Abstract

Background: Fungemia caused by non-*Candida* yeasts is rare but represents an emerging clinical problem that remains less well recognized and studied. These organisms often exhibit intrinsic resistance or reduced susceptibility to commonly used empirical antifungal agents, such as fluconazole and echinocandins. This poses significant challenges for empirical antifungal therapy. Objectives: To describe the clinical characteristics, antifungal treatments, and outcomes of pediatric patients with bloodstream infections due to non-*Candida* yeasts and to summarize the antifungal susceptibility profiles of available isolates. Methods: This retrospective study reviewed all episodes of fungemia caused by non-*Candida* yeasts at a tertiary pediatric center between 1 January 2020 and 1 September 2025. Results: Of the 139 yeast-related fungemia episodes identified during the study period, five (3.6%) were caused by non-*Candida* yeasts: three by *Trichosporon* spp., one by *Rhodotorula mucilaginosa*, and one by *Magnusiomyces clavatus* (formerly *Saprochaete clavatus*). Two cases occurred as breakthrough infections under ongoing antifungal treatment. Empirical antifungal treatments most often included amphotericin B, fluconazole, or echinocandins. The median time to species-level identification after the first positive culture result was six days (range 4–7), highlighting a considerable delay that may critically affect clinical management. Overall mortality was 40%, while attributable mortality due to non-*Candida* fungemia was 20%. Conclusions: Non-*Candida* yeasts, although infrequent, represent clinically important pathogens in pediatric fungemia due to their potential resistance to standard empirical antifungal agents. Early species-level identification and awareness of expected susceptibility patterns are essential to guide appropriate initial therapy and improve outcomes.

## 1. Introduction

Invasive fungal infections (IFIs) predominantly affect immunocompromised or critically ill patients and remain an important cause of morbidity and mortality in children [1,2,3]. Yeast-related fungemia represents a substantial proportion of IFIs, with *Candida* species accounting for most pediatric cases [4,5,6]. In contrast, fungemia caused by non-*Candida* yeasts is uncommon; however, recent reports indicate an increasing frequency, particularly among patients with hematologic malignancies, prolonged neutropenia, intensive care hospitalization, or exposure to broad-spectrum antimicrobials [1,2,3,5]. Non-*Candida* yeasts are widely distributed in the environment and may colonize skin and mucosal surfaces. Although sometimes regarded as contaminants, these organisms can cause life-threatening infections in susceptible hosts [7,8].

Several non-*Candida* yeast genera have been reported as opportunistic human pathogens, including *Trichosporon*, *Rhodotorula*, *Saprochaete/Magnusiomyces*, *Malassezia*, *Saccharomyces*, *Cryptococcus*, and *Geotrichum* [2,3,4]. Although these organisms are less frequently encountered non-*Candida* species, they can cause severe bloodstream infections in immunocompromised hosts and are often associated with distinct antifungal susceptibility patterns. Their emergence has been attributed to several factors, including improved survival of immunocompromised children, extensive use of antifungal agents, central venous catheter dependence, broad-spectrum antibiotic exposure, and intensive chemotherapy or immunosuppressive therapies [3,4,5,6,7,8]. Despite growing recognition, epidemiological data on these rare pathogens, particularly in pediatric populations, remain limited. Clinical management is further complicated by diagnostic delays, heterogeneous antifungal susceptibility profiles, absence of standardized clinical breakpoints, and uncertainty regarding optimal therapeutic strategies [1,3]. For these reasons, non-*Candida* yeast fungemia warrants focused clinical attention.

Given the rarity of these infections in children, the present study was designed as a retrospective descriptive observational study aiming to characterize the clinical features, microbiological findings, antifungal management, and outcomes of rare non-*Candida* yeast fungemia episodes observed at our tertiary pediatric center over a six-year period.

## 2. Patients and Methods

### 2.1. Collection of Patient Data

This study was designed as a retrospective descriptive observational study conducted at a tertiary pediatric referral center. Between 1 January 2020 and 31 December 2025, we retrospectively reviewed bloodstream infections caused by yeast species other than *Candida*. Pediatric patients (<18 years) with at least one positive peripheral or catheter-derived blood culture yielding a rare non-*Candida* yeast were included.

Yeast species historically classified within the genus *Candida* but later reassigned to other genera (e.g., *Clavispora lusitaniae*, *Meyerozyma guilliermondii*, *Yarrowia lipolytica*, *Cyberlindnera fabianii*, *Wickerhamomyces anomalus*, and *Wickerhamiella pararugosa*) were excluded to avoid taxonomic ambiguity [8].

Electronic medical records were reviewed for demographic data, underlying conditions, central venous catheter use, recent antimicrobial exposure, total parenteral nutrition, corticosteroid therapy, neutropenia duration, intensive care stay, antifungal treatment, clinical response, and mortality outcomes.

### 2.2. Definitions

Rare yeast fungemia was defined as isolation of non-*Candida* yeasts from at least one blood culture in patients with clinical signs of infection. Neutropenia was defined as an absolute neutrophil count < 500 cells/µL. Previous antimicrobial exposure was defined as ≥3 days of therapy within 30 days prior to fungemia. Breakthrough IFIs were defined as infections occurring during antifungal prophylaxis or treatment [9].

### 2.3. Identification of Isolates

Between 2020 and 2023, blood cultures were processed using the BACT/ALERT system (bioMérieux, Marcy-l’Étoile, France), and from 2023 onwards, the BD BACTEC system (Becton Dickinson, Franklin Lakes, NJ, USA) was used. Isolates were identified using matrix-assisted laser desorption/ionization time-of-flight mass spectrometry (MALDI-TOF MS). Between 2020 and 2023, microbial identification was performed using the VITEK MALDI-TOF MS system (bioMérieux, Marcy-l’Étoile, France), and from 2023 onwards, isolates were identified with the MALDI Biotyper system (Bruker Daltonics, Bremen, Germany). The system was routinely calibrated according to the manufacturer’s instructions. For isolates with low-confidence scores, conventional biochemical tests were performed for confirmation.

Once a blood culture signaled positive for yeast, isolates were subcultured on appropriate media. Species identification was performed after adequate colony growth using MALDI-TOF MS. Accordingly, the time to species identification reflected the entire diagnostic workflow, including blood culture positivity, subculture growth, and colony preparation prior to MALDI-TOF analysis.

This study was approved by the Ethics Committee of Ankara University (Decision number 2025/746).

### 2.4. Informed Consent Statement

Informed consent was waived due to the retrospective nature of the study. The study involved analysis of anonymized clinical data collected during routine clinical care, and no additional interventions were performed on the patients.

*Statistical Analysis***.** Statistical analyses were performed using IBM SPSS Statistics for Windows, version 25.0 (IBM Corp., Armonk, NY, USA). Continuous variables were expressed as median with range (minimum–maximum) due to the non-normal distribution of most parameters. Categorical variables were summarized as absolute counts and percentages.

## 3. Results

During the study period, non-*Candida* yeast fungemia was identified in five patients (three *Trichosporon* spp., one *Magnusiomyces* [*Saprochaete*] *clavatus*, and one *Rhodotorula mucilaginosa*). Among 139 yeast-related fungemia episodes recorded at our center, rare non-*Candida* yeasts accounted for 3.6%. Demographic characteristics, underlying conditions, antifungal therapy, and clinical outcomes are summarized in Table 1.

Case 1: A 9-year-old girl diagnosed with unilateral sporadic retinoblastoma presented on the 12th day of the sixth cycle of ifosfamide, carboplatin, and etoposide (ICE) therapy with a fever reaching 39 °C in the axillary region. Physical examination findings were normal. The whole blood count revealed a hemoglobin level of 10.9 g/dL, a white blood cell (WBC) count of 260/mm^3^, a neutrophil count of 40/mm^3^, and a platelet count of 157.000/mm^3^. The C-reactive protein (CRP) was 7.2 mg/L. Blood cultures were obtained from catheter and peripheral blood, and urine cultures were obtained, and intravenous (IV) piperacillin-tazobactam treatment was initiated for febrile neutropenia. The patient tested positive for SARS-CoV-2 in a respiratory multiplex polymerase chain reaction (PCR) and was placed in isolation.

During follow-up, yeast signals were detected in the peripheral blood culture at the 32nd hour, prompting control cultures to be taken and intravenous (IV) caspofungin treatment to be initiated. However, on the sixth day of hospitalization, *T. asahii* was detected in the peripheral blood culture, and IV voriconazole therapy was initiated instead of caspofungin. Piperacillin-tazobactam therapy was also discontinued on the same day. In response to treatment, the patient’s fever subsided, her clinical condition stabilized, and neutropenia recovered gradually during antifungal therapy. The patient was discharged on the sixth day of IV therapy with oral voriconazole, and the treatment period was completed in three weeks.

**Figure 1:**
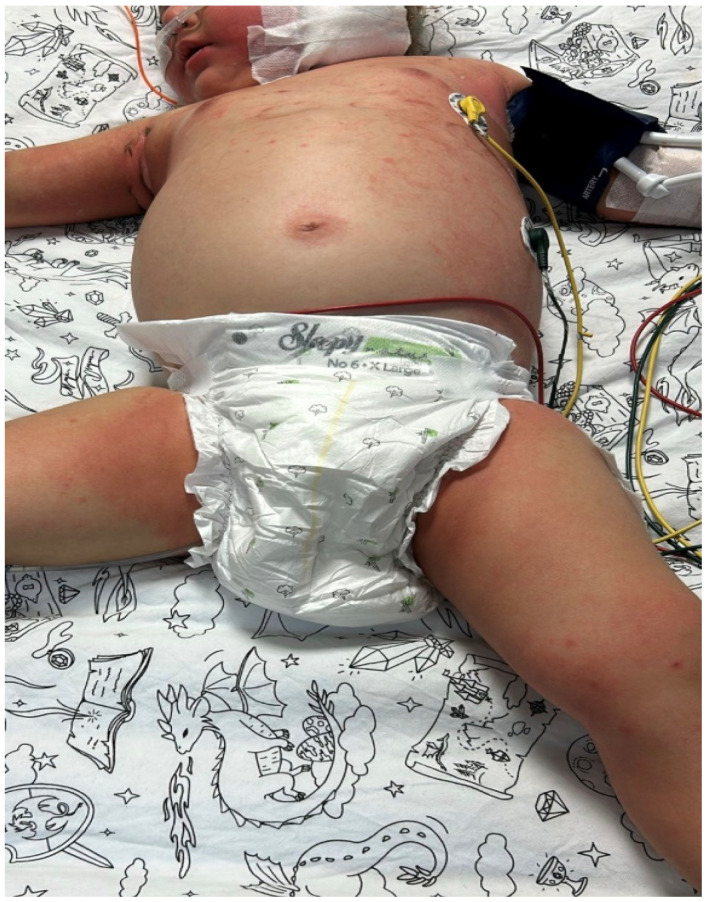
Anterior trunk and thighs showing scattered macular lesions on an erythematous background, with accentuated erythema in flexural areas. Device-related marks from monitoring/probes are visible.

**Figure 2:**
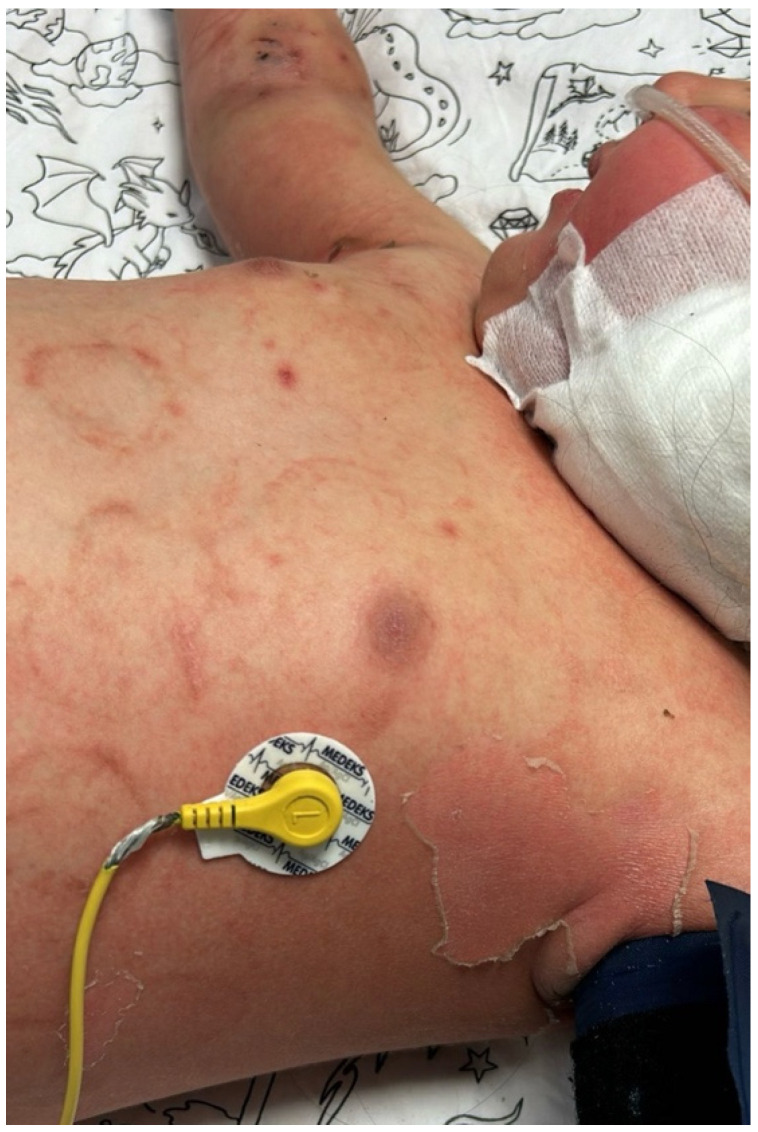
Lamellar superficial desquamation in the left axillary region and macular rash on the anterior trunk.

Case 2: A 23-month-old girl with newly diagnosed acute myeloid leukemia (AML) developed febrile neutropenia on the 10th day of induction chemotherapy. Piperacillin-tazobactam therapy was initiated. As the fever persisted, antibiotic therapy was gradually expanded and revised to include IV meropenem, teicoplanin, liposomal amphotericin B, levofloxacin, and colistin. On the sixth day of fever, she was admitted to the pediatric intensive care unit with a diagnosis of sepsis, where high-flow oxygen support was initiated and the central catheter was removed. On the seventh day of fever, macular rashes developed with widespread erythema on the trunk, extremities, and scalp (Figure 1 and Figure 2). Viral serology and PCR tests were performed, and intravenous immunoglobulin (IVIG) therapy was initiated. A biopsy was taken due to persistent skin lesions. A yeast signal was detected in the blood culture at 29 h, and caspofungin was started in addition to the existing liposomal amphotericin B therapy.

Plasmapheresis (PEX) was performed on the tenth day of fever due to the emergence of hyperinflammatory findings. With the development of renal failure, continuous renal replacement therapy (CRRT) was initiated. On the eleventh day of fever, the patient was intubated, and inotropic support and hydrocortisone therapy were added. On the 12th day of fever, the fifth day of blood culture, *T. asahii* was reported as the causative agent. Hence, caspofungin treatment was discontinued, and voriconazole treatment was added to liposomal amphotericin B (3 mg/kg/d) treatment. During this process, a thoracoabdominal computed tomography (CT) revealed diffuse peribronchovascular reticular infiltration and ground-glass opacities in both lungs, along with diffuse infiltration in the spleen and newly developed widespread millimetric hypodense foci. The findings were considered consistent with the underlying malignancy and, in particular, systemic fungal infection. No focus of infection was detected on echocardiography. During follow-up, peritoneal sampling was performed due to the development of abdominal distension and suspicion of perforation. *T. asahii* growth was detected in peritoneal fluid culture, but the causative agent could not be isolated in tissue culture. The skin biopsy was considered nonspecific; immunohistochemical staining did not reveal findings suggestive of leukemic infiltration or fungal infection. Although the cause of the rash could not be definitively established, the temporal association with fungemia suggested potential clinical relevance.

Despite dual antifungal therapy, intermittent growth in blood cultures persisted for approximately 20 days. No new growth was detected during the following 45-day period, and abdominal imaging revealed regression of splenic involvement. During follow-up, thrombocytopenia secondary to the underlying malignancy and recurrent septic episodes led to intracranial hemorrhage, resulting in hydrocephalus. Therefore, a ventriculoperitoneal shunt was placed. The patient’s neurological condition subsequently deteriorated, and she developed brain death and died.

Case 3: A 27-year-old mother gave birth to a male neonate weighing 3450 g at 39 weeks via cesarean section. The infant presented to an external center on the second postnatal day with complaints of failure to feed and lethargy. The patient was evaluated with preliminary diagnoses of early neonatal sepsis or metabolic disease, and ornithine transcarbamylase (OTC) deficiency was considered the most likely cause. He was transferred to our neonatal intensive care unit on the fourth day.

Upon admission, he was in poor general condition, intubated, with widespread edema and abdominal distension. There was a necrotic decubitus ulcer 3 cm in diameter on his back. Blood ammonia level was measured as 1199 µmol/L. A hemodialysis catheter was inserted, and CRRT was initiated. Blood and urine cultures were obtained, and treatment with IV meropenem and vancomycin, which had been started at the external center, was continued.

On the third day of follow-up, IV amikacin and liposomal amphotericin B (3 mg/kg/d) were added to the treatment due to clinical deterioration. A paracentesis was performed on the patient, who had widespread ascites; leukocytes were detected at 67,161/mm^3^ and erythrocytes at 15/mm^3^ in the peritoneal fluid. Treatment was readjusted to IV meropenem infusion, colistin, and vancomycin. However, despite all inotropic, antibiotic, and supportive treatments, the patient died on the seventh day of hospitalization (the fourth day of liposomal amphotericin B treatment).

After death, yeast signals were reported in the blood culture taken 95 h before starting liposomal amphotericin B treatment. The causative agent was identified as *Trichosporon* spp. on the sixth day of typing. Concurrent peritoneal cultures grew *Klebsiella pneumoniae* (susceptible to carbapenems and amikacin), *Stenotrophomonas maltophilia*, and *Enterococcus faecalis*. Due to the patient’s death, advanced species identification and antifungal susceptibility testing could not be performed on the *Trichosporon* isolate. *Trichosporon* was also detected in postmortem blood, catheter, and cerebrospinal fluid samples.

Case 4: A 1.5-month-old boy was transferred to our hospital’s pediatric intensive care unit with preliminary diagnoses of subdural hematoma, subarachnoid hemorrhage, and pulmonary contusion following a car accident. The patient was intubated and empirically treated with IV ceftriaxone, which was discontinued on the 7th day. On the 10th day of hospitalization, peripheral blood, urine, and catheter blood cultures were obtained due to the development of fever, and empirical piperacillin-tazobactam treatment was initiated. As the fever persisted, *Pseudomonas aeruginosa* growth was detected in the daily peripheral and catheter blood cultures. Consequently, treatment was revised to IV meropenem and amikacin, and the catheter was removed. Empirically, IV fluconazole therapy was added due to persistent fever. A yeast signal was reported at the 96th hour of the blood culture. Subsequently, on the 7th day, *R. mucilaginosa* was reported in the blood culture, and fluconazole was discontinued. Treatment was changed to IV liposomal amphotericin B. Meropenem and amikacin treatments were discontinued on day 12. Liposomal amphotericin B treatment was completed on day 14. No pathology was detected in the cardiology evaluation, and there was no growth in the follow-up cultures. The patient was discharged in a clinically stable condition.

Case 5: An 11-year-old girl diagnosed with relapsed B-acute lymphoblastic leukemia (ALL) presented to the emergency department with febrile neutropenia. Piperacillin-tazobactam therapy was initially started. Upon physical examination, tenderness was detected in the right lower quadrant, and IV metronidazole and caspofungin were added to the treatment with a preliminary diagnosis of typhlitis. Abdominal ultrasound showed hepatosplenomegaly, minimal free fluid in the pelvis, moderate wall thickening, and subserosal edema in the cecum and terminal ileum. Due to persistent fever, piperacillin-tazobactam was replaced with meropenem, and teicoplanin was added to the treatment due to the presence of oral mucosal lesions. The patient’s fever persisted during follow-up, and *Salmonella* spp. growth was detected in the catheter blood culture. Intravenous ciprofloxacin and catheter lock therapy were initiated. Clinical improvement was achieved after this treatment, and the fever subsided. However, on the 10th day of treatment, the fever rose again, and tachypnea and desaturation developed. Physical examination revealed decreased bilateral breath sounds, particularly prominent in the lower right lung zone. An emergency chest CT scan showed ground-glass opacities in the bilateral lower zones. The catheter was removed; IV liposomal amphotericin B (3 mg/kg/d) and trimethoprim-sulfamethoxazole were added to the treatment, and caspofungin and metronidazole were discontinued. Serum galactomannan assay and *Pneumocystis jirovecii* PCR tests were negative. A minimal amount of pericardial fluid was detected on cardiac examination.

On the fourth day of liposomal amphotericin B therapy, yeast signals were reported in peripheral and catheter blood cultures. Upon identification of the causative agent as *M. clavatus*, IV voriconazole was added to the treatment on the fifth day. On the 10th day of amphotericin therapy and the sixth day of voriconazole, the patient developed nonspecific, petechiae-like erythematous rashes. A biopsy could not be performed on this patient, who had severe thrombocytopenia and was receiving palliative care. Because of persistent growth in blood cultures, the dosage of liposomal amphotericin B was increased to 5 mg/kg/day. On the second day of the dosage increase (11th day of amphotericin and 7th day of voriconazole), the fever subsided, and the rash completely disappeared within seven days. After 72 h without fever, antibiotics were discontinued; the patient was monitored under liposomal amphotericin B and voriconazole treatment. On the 11th day of voriconazole treatment, the patient developed blurred vision. No papilledema was detected on fundus examination, and no pathology was observed on cranial imaging. However, as the complaints persisted and no other cause was found, treatment was switched to posaconazole on day 15, considering that voriconazole might be a common side effect. Antifungal treatment was completed in 21 days. Liposomal amphotericin B treatment was continued for another week and was discontinued on day 35, after the patient recovered from neutropenia and a thoracic CT showed regression of the lesions.

### General Findings

Of the five patients included in the study, three (60%) had underlying hematologic or oncologic diseases and one (20%) had suspected metabolic disease. Sixty percent of patients had a history of neutropenia lasting a median of 12 days (range 4–13) prior to the onset of fungemia. Sixty percent of patients were monitored in the neonatal or pediatric intensive care unit, and 80% had a central venous catheter. No patient had a history of surgical intervention within the last month. The median time between hospital admission and onset of fungemia was 12 days (range 5–21).

Yeast growth was detected in blood cultures in all cases. The median time to a positive fungal signal was 32 h (range 29–96). Species were identified in four patients; in one fatal case (Case 3), identification was completed postmortem, and susceptibility testing was not performed. Across the cohort, identified species comprised *Trichosporon* spp. (*n* = 3), *R. mucilaginosa* (*n* = 1), and *M. clavatus* (*n* = 1).

Antifungal susceptibility testing could only be performed for two isolates (1 *T. asahii*, 1 *M. clavatus*). In the remaining cases, susceptibility testing could not be performed because viable colonies were not available for further testing or because the patient died shortly after culture positivity. Since clinical breakpoints have not yet been established for these species, only minimum inhibitory concentration (MIC) distributions were reported. Voriconazole and itraconazole showed in vitro activity against both isolates with low MIC values in the range of 0.03–0.25 µg/mL, while relatively high MIC values (2–4 µg/mL) were detected for fluconazole. Although amphotericin B showed a low MIC value (0.5 µg/mL) for *T. asahii*, clinical efficacy could not be achieved, and voriconazole was added to the treatment regimen in the relevant case.

Upon initial yeast positivity—before species identification and susceptibility results—empirical therapy was initiated or adjusted according to each patient’s on-treatment status: fluconazole (*n* = 1), liposomal amphotericin B (*n* = 1), and echinocandin (*n* = 2); one patient died on the day culture positivity was reported. After species identification and/or susceptibility data became available, antifungal regimens were modified in all surviving cases.

Breakthrough fungemia occurred under liposomal amphotericin B (*n* = 1) and under echinocandin therapy (*n* = 1). Species distribution, minimum inhibitory concentration (MIC) values, preferred antifungal therapies, and clinical outcomes are summarized in Table 2.

Overall mortality was 40%, and attributable mortality was 20%. In the attributable death, the interval from fungemia onset to death was 4 days.

## 4. Discussion

In this retrospective descriptive study, rare non-*Candida* yeasts were infrequently encountered over a six-year period (five cases), yet these infections remained clinically significant because of delayed species-level identification and limited antifungal susceptibility data. Given the rarity of these pathogens and the small number of cases, the findings of our study should be interpreted primarily as descriptive clinical observations rather than definitive epidemiological conclusions. Similarly to our experience, most available data on rare non-*Candida* yeast infections in children derive from small case series or individual case reports due to the low incidence of these pathogens in pediatric populations [10,11,12,13,14]. Nevertheless, our experience highlights important diagnostic and therapeutic challenges associated with these infections in vulnerable pediatric patients.

In our series, empirical antifungal regimens frequently required modification after species identification, underscoring the gap between empiric strategies primarily targeting *Candida* spp. [15] and the intrinsic resistance or reduced susceptibilities typical of non-*Candida* yeasts [16]. Timely and appropriate antifungal therapy is pivotal in fungemia, as therapeutic delay or suboptimal initial choices are associated with increased mortality [8,16]. In our cohort, the median time from blood culture positivity to species-level identification was six days. Although MALDI-TOF MS allows rapid species identification once an adequate colony is available, the reported identification time reflects the entire diagnostic workflow rather than the MALDI-TOF procedure itself. This interval includes the time required for blood culture positivity and subculture growth before MALDI-TOF analysis can be performed. In addition, routine laboratory workflow factors such as processing schedules, weekends, and laboratory staffing patterns may occasionally contribute to delays between culture positivity and final species identification. Therefore, clinicians should be aware of predisposing risk factors and maintain a high index of suspicion for these uncommon pathogens [8]. In our cohort, central venous catheter use and recent antibiotic exposure were each present in 80% of cases, while neutropenia was observed in 60%. Consistent with prior reports, well-recognized risk factors—including hematologic malignancy, prolonged neutropenia, intensive care unit admission, central venous catheter use, and recent broad-spectrum antibiotic exposure—were prevalent in our cohort [7,16]. Similar predisposing factors have been described in pediatric studies of rare yeast infections, where central venous catheter use, prolonged hospitalization, exposure to broad-spectrum antibiotics, and underlying immunocompromising conditions are frequently observed [13]. Overall, the epidemiology of rare non-*Candida* yeast fungemia in children appears to mirror that of invasive candidiasis, predominantly affecting immunocompromised patients, particularly those with hematologic malignancies, prolonged neutropenia, and central venous catheter dependence.

After assessing risk factors, it is necessary to focus on the clinical significance of specific yeast species. *Trichosporon* spp. are rare yeast species that can colonize the skin, gastrointestinal, and respiratory systems, causing superficial and invasive infections. *T. asahii* is the primary causative agent of invasive trichosporonosis, and hematologic malignancy patients and premature newborns are particularly at risk. Azoles are the first-line agents for treatment [2]. *Trichosporon* spp. are naturally resistant to echinocandins and show variable susceptibility to amphotericin B [7]. Our series included three cases of *Trichosporon* spp.: In one case, despite a low MIC value, breakthrough infection developed under amphotericin B, and a response was achieved with the addition of voriconazole (Case 2). In a fatal case, species identification was only possible postmortem, and susceptibility testing could not be performed (Case 3). In another case, although susceptibility testing could not be performed, clinical improvement was achieved with empirical voriconazole (Case 1). Metastatic skin lesions, pneumonia, and spleen and liver abscesses are frequently reported clinical findings in the literature [8,17]. In our series, skin and spleen involvement with pneumonia was observed in one patient, while peritonitis and central nervous system involvement with simultaneous cerebrospinal fluid culture growth were observed in a newborn. The prognosis for trichosporonosis is generally poor; mortality has been reported to be 28.5–30% in children and 80–87.5% in adults [10]. Pediatric studies further highlight the clinical severity of invasive trichosporonosis. In a recent 10-year single-center pediatric study, 12 cases of invasive *Trichosporon* infection were reported, with an overall mortality rate of 41.7%, emphasizing the significant morbidity and mortality associated with these infections in immunocompromised children [13]. Mortality in our cases was 66.7%, which appears higher than the pediatric rates reported in the literature; however, this finding should be interpreted cautiously, given the very small sample size and the severity of underlying conditions in our patients.

Another rare yeast species is *Rhodotorula*. These yeasts are commonly found in the environment and can colonize the skin and mucous membranes. The most frequently isolated species is *R. mucilaginosa*, which is a causative agent of opportunistic infections, particularly in immunocompromised patients. The most important predisposing factors are CVC and malignancy. *Rhodotorula* species are naturally resistant to echinocandins and fluconazole. Liposomal amphotericin B and/or flucytosine are recommended for treatment [2]. Although susceptibility testing could not be performed in this case, the patient responded well to liposomal amphotericin B treatment.

Another important group of factors is *Saprochaete/Magnusiomyces* species. These pathogens have been identified as causative agents of infection, particularly in cases of hematological malignancy, chemotherapy, prolonged neutropenia, and CVC [7,8]. Chemotherapies that compromise the mucosal barrier’s integrity can lead to gastrointestinal translocation and fungemia development [11]. Pediatric data on these infections remain extremely limited, and only a small number of cases have been reported in children. A pediatric case of *M. clavatus* fungemia occurring after hematopoietic stem cell transplantation highlighted prolonged neutropenia and severe immunosuppression as key predisposing factors and emphasized the importance of antifungal therapy combined with immune reconstitution for successful infection control [14]. In this case, the previous *Salmonella* bacteremia and gastrointestinal involvement were thought to pave the way for fungemia. Clinically, it usually presents with a disseminated infection pattern. Most isolates appear resistant to echinocandins and fluconazole but sensitive to amphotericin B, flucytosine, and newer azoles [11]. Therefore, a combination of voriconazole, posaconazole, amphotericin B, and flucytosine is recommended for treatment. However, despite these treatments, mortality rates as high as 60 to 85% have been reported in some studies [11,12].

Implications for practice. Given the mismatch between standard empiric regimens for candidemia and the susceptibility profiles of non-*Candida* yeasts, our findings also have important implications for empirical antifungal therapy in high-risk pediatric patients. Because standard empiric regimens frequently rely on echinocandins or fluconazole, they may not provide adequate coverage for certain rare yeasts such as *Trichosporon* or *Rhodotorula* species, which may exhibit intrinsic resistance or reduced susceptibility to these agents. Where susceptibility testing is limited or delayed, risk-factor assessment and species-directed empiric adjustments may improve outcomes. Empiric antifungal algorithms should be tailored to local species and susceptibility patterns, and laboratories should immediately alert clinicians of any yeast-positive blood culture.

Limitations: The primary limitation of this study is the small cohort size (*n* = 5), which restricts the generalizability of the findings and prevents robust statistical analysis. The retrospective, single-center design introduces potential selection biases. Additionally, antifungal susceptibility testing could not be performed for all isolates, limiting assessment of resistance patterns and their relationship to clinical outcomes. Larger, multicenter studies with standardized diagnostic workflows are needed to better define the epidemiology and optimize treatment strategies for these rare pathogens.

In conclusion, although non-*Candida* yeasts account for only a small proportion of fungal infections, they are clinically significant due to their antifungal resistance and high mortality rates. In settings where species-level identification and susceptibility testing take days, initial empiric therapy should be guided by local epidemiology and patient risk factors, then promptly individualized once organism- and susceptibility-level data become available. Strengthening rapid diagnostic capacity and surveillance will improve both empiric decision-making and patient-level outcomes, recognizing the broad clinical spectrum of these infections and the need for tailored care.

## Figures and Tables

**Table 1:** Demographic and clinical characteristics of patients with fungemia (*n* = 5) caused by rare non-*Candida* yeasts.

Patient	Case 1	Case 2	Case 3	Case 4	Case 5
Age (mo/d)	103 mo	24 mo	4 d	1.5 mo	132 mo
Underlying condition	Retinoblastoma	Acute myeloid leukemia	Metabolic disease	Trauma	Relapse B-ALL
Clinical Findings	Fever	Fever, rash, abdominal distension and tenderness	Fever, abdominal distension and tenderness, edema	Fever	Fever, rash, pneumonia
Concomitant virus	COVID-19	-	-	-	-
Concomitant bacteria	-	*Staphylococcus haemolyticus* (blood culture)	*Klebsiella pneumoniae*, *Stenotrophomonas maltophilia*, *Enterococcus faecalis* (peritoneal culture)	*Pseudomonas aeruginosa*	-
Pediatric intensive care unit (PICU) admission	N	Y	Y	Y	N
Length of PICU stay (d)	-	67	7	21	-
Length of hospital stay (d)	12	86	7	32	180
Antibiotic treatment	Piperacillin–tazobactam	Meropenem, levofloxacin, linezolid, colistin	Meropenem, vancomycin, amikacin, colistin	Meropenem, amikacin	Meropenem, ciprofloxacin, trimethoprim–sulfamethoxazole, vancomycin
Duration of hospital stay in the last 30 days until the onset of fungemia (d)	5	21	7	13	12
Time to yeast positivity (h)	32 h	29 h	95 h	96 h	32 h
Species identification (time to identification, d)	*Trichosporon asahii* (6 d)	*Trichosporon asahii* (5 d)	*Trichosporon* spp. (6 d)	*Rhodotorula mucilaginosa* (7 d)	*Magnusiomyces (Saprochaete) clavatus* (4 d)
Time to antifungal susceptibility results (days)	-	6 d	-	-	4 d
Use of central venous catheter	-	+ (hemodialysis catheter)	+ (hemodialysis catheter)	+	+
Neutropenia duration (d)	4	13	-	-	12
Total parenteral nutrition	-	-	+	-	-
Prior antimicrobial exposure	-	+	+	+	+
Prior chemotherapy	+	+	-	-	+
Use of corticosteroids	-	+	-	-	+
Breakthrough fungemia	-	+	-	-	+
Organ involvement	-	Gastrointestinal disease (spleen has several millimeter lesions, suspected intestinal perforation), pneumonia, cutaneous involvement	Gastrointestinal and central nervous system disease	-	Gastrointestinal, cutaneous, respiratory disease
Empirical antifungal therapy	CAS	CAS + L-AMB	L-AMB	FLU	L-AMB
Target antifungal therapy	VOR	VOR + L-AMB	-	L-AMB	VOR (POS) + L-AMB
Treatment duration (d)	21	60 (VOR), 46 (L-AMB)	4	14	15 (VOR) → 6 (POS), 35 (L-AMB)
Outcome	Cure	Died (non-fungemia-attributable mortality)	Died	Cure	Cure

B-cell acute lymphoblastic leukemia: B-ALL, caspofungin: CAS, coronavirus Disease 2019: COVID-19, day: d, fluconazole: FLU, liposomal amphotericin B: L-AMB, months: mo, posaconazole: POS, voriconazole: VOR.

**Table 2:** Species distribution, antifungal therapy, and outcomes of patients with breakthrough fungemia.

Patient	Antifungal Drug Under Which Breakthrough Fungemia Was Developed	Yeast Species Responsible for Breakthrough Fungemia	MIC Values (µg/mL)						Antifungal Drug for the Treatment of Breakthrough Fungemia	Outcome of Patients with Breakthrough Fungemia
			L-AMB	CAS	FLU	VOR	POS	ITRA		
Case 2	L-AMB	*Trichosporon asahii*	0.5	-	4	0.06	-	0.25	VOR and L-AMB	Died (non-fungemia-attributable mortality)
Case 5	CAS	*Magnusiomyces (Saprochaete) clavatus*	0.25	>8	2	0.03	0.25	0.06	VOR(POS) and L-AMB	Cure

Abbreviations: CAS, caspofungin; FLU, fluconazole; ITRA, itraconazole; POS, posaconazole; VOR, voriconazole; L-AMB, liposomal amphotericin B; MIC, minimum inhibitory concentration.

## Data Availability

The original contributions presented in the study are included in the article. Further inquiries can be directed to the corresponding author.
